# Unilateral Mastication Evaluated Using Asymmetric Functional Tooth Units as a Risk Indicator for Hearing Loss

**DOI:** 10.2188/jea.JE20180052

**Published:** 2019-08-05

**Authors:** Joo-Young Lee, Eun-Song Lee, Gyung-Min Kim, Hoi-In Jung, Jeong-Woo Lee, Ho-Keun Kwon, Baek-Il Kim

**Affiliations:** 1 Department of Preventive Dentistry & Public Oral Health, Brain Korea 21 PLUS Project, Yonsei University College of Dentistry, Seoul, Republic of Korea; 2 Oral Science Research Institute, Yonsei University College of Dentistry, Seoul, Republic of Korea

**Keywords:** epidemiology, functional tooth units, hearing loss, oral health, unilateral mastication

## Abstract

**Background:**

Some previous studies reported hearing ability can be reduced by impaired masticatory ability, but there has been little evidence reported of an association between hearing loss and unilateral mastication. Therefore, this study aimed to investigate the relationship between unilateral mastication (UM), estimated from individual functional tooth units (FTUs), and hearing loss in a representative sample of Korean adults.

**Methods:**

The analyzed data were obtained from 1,773 adults aged 40–89 years who participated in Korean national survey. Hearing loss was defined as a pure-tone average of >25 dB at frequencies of 0.5, 1, 2, and 4 kHz in either ear. In each subject, UM was calculated as the difference in the sums of the FTU scores, which is an index of posterior tooth occlusion, on the two sides of the oral cavity. The scores were used to classify the UM into low, moderate, and high. The adjusted odds ratios (aORs) and their 95% confidence intervals (CIs) were calculated in multivariable logistic regression analyses.

**Results:**

When controlling for sociodemographic factors, the aOR for hearing loss was 3.12 (95% CI, 1.21–8.03) for high UM relative to low UM. This association remained in a fully-adjusted model containing factors related to noise exposure (aOR 2.88; 95% CI, 1.12–7.46).

**Conclusion:**

Adults with high UM as measured using FTUs showed a higher occurrence of hearing loss than those with low UM.

## INTRODUCTION

The World Health Organization (WHO) has estimated that almost 15% of adults worldwide have some degree of hearing loss.^1^ Hearing loss is often overlooked in the early stages of the disease due to the subtlety of the symptoms. Furthermore, many older adults accept that hearing loss is inevitable. However, the severity of hearing loss among those of the same age varies with the individual sensitivity, and the risk of hearing loss is associated with being male, smoking, and having lower education, military service, industrial employment, and noise exposure.^2^^–^^4^ Mild hearing loss can also lead to symptoms of depression and social isolation due to its adverse effects on verbal communication.^5^^,^^6^ In addition, since presbycusis is rarely reversible, it is necessary to identify the hearing status in adults in order to avoid progressive hearing loss.^7^

An association between dentate status and hearing loss has been reported. Previous studies suggest that hearing ability can be reduced by temporomandibular disorder (TMD),^8^ tooth loss,^9^^,^^10^ and impaired masticatory ability.^11^^–^^13^ Initially, an association between hearing loss and tooth loss was reported.^9^ Later, clinical evidence has shown that hearing can be restored through the removal of unilateral chewing habits via prosthodontic treatment.^11^^,^^13^ With respect to this connection, one experimental study found that one side of the maximum occlusal pressure affects the auditory-evoked magnetic fields.^11^ However, there has been little epidemiologic evidence reported of an association between tooth loss and hearing loss,^9^ and to our knowledge, no epidemiologic evidence of an association between hearing loss and UM.

One important aspect of UM is balancing of oral status. Preliminary research has found UM to be more common in individuals with an unequal distribution of residual teeth on both sides of the mouth than in those with an even distribution.^14^ Therefore, the individual UM level can be estimated based on the difference in functional tooth units (FTUs). FTUs have been used as a masticatory ability index to evaluate the oral condition or dietary intake.^15^^–^^18^ FTUs was developed from the concept of occluding pairs (OPs) and defined as pairs of opposing posterior teeth—premolars and molars. A lack of FTUs is considered to be a key factor contributing to the loss of masticatory ability.^16^ The relationship between OPs and hearing loss had been examined previously,^10^ but, to our knowledge, no study has assessed the relationship between FTUs and auditory threshold. In addition, this approach on asymmetric FTUs can make it possible to compare changes in auditory thresholds according to the UM level. Therefore, the aim of this study was to determine the relationship between the UM level, estimated from individual FTUs, and hearing loss in a representative sample of Korean adults.

## MATERIALS AND METHODS

### Study population

The Korean National Health and Nutrition Examination Survey (KNHANES) is conducted to evaluate the health and nutritional status. It is a nationwide, multistage, and stratified survey of a representative sample of the South Korean population. This survey has been performed in a 3-year cycle since 1998, and it produces statistical information on health indicators requested by international organizations, such as the Organization for Economic Cooperation and Development (OECD) or WHO, that allows comparisons between countries. The data used in the present study constituted a subset of the data obtained in KNHANES 2010–2012, and it was necessary to determine periodontal status by assessing the masticatory ability using only a dental formula. We only used data from 2010 and 2012, since oral examination data for the community periodontal index (CPI) were not published in 2011. The initial sample size was 7,014 adults aged 40–89 years who completed oral and audiometric examinations.

The key considerations when assessing the masticatory ability were factors related chewing impairment—periodontal disease and untreated caries. For the present analysis, we excluded participants with a CPI score of 4 (*n* = 2,300), since the advanced periodontal status can reportedly reduce the masticatory ability due to pain and teeth mobility.^19^^,^^20^ Second, we excluded an additional 2,037 participants who had artificial teeth on implant-supported, fixed (bridge pontics) and removable prostheses, since we obtained limited information only about the presence or absence of prostheses in each upper or lower jaw from national survey data. Additionally, 412 participants with decayed teeth (DT) were excluded, because it was difficult to confirm severity and pain/symptoms of DT in this study. Moreover, in the KNHANES dataset, information on the dental treatment needs (TN) of individual teeth is also available. Especially, TN codes 6–8 indicate needs for extraction-related severe caries, periodontitis, and so on. These conditions make it difficult to chew normally, so participants who had TN codes 6–8 were excluded (*n* = 272). Finally, those with external ear disease (*n* = 220) were excluded, leaving a total of 1,773 subjects for inclusion in the analysis.

The data set produced by KNHANES is publicly available, and the study protocol was reviewed and approved by the Institutional Review Board of the Korea Centers for Disease Control and Prevention. All of the included individuals signed informed-consent forms before participation.

### Audiometric measurement

Pure-tone audiometric testing was conducted by trained otolaryngologists in a sound-proof booth using an audiometer. The otolaryngologist provided instructions to each participant regarding how the automated hearing test is performed, and measured the air-conduction thresholds. The frequencies tested were 0.5, 1, 2, 3, 4, and 6 kHz. Hearing loss was defined as a pure-tone average (PTA) of >25 dB, and the 4-PTA was calculated at frequencies of 0.5, 1, 2, and 4 kHz in either ear, which is consistent with the definition used by the WHO.^21^

### Quantification of unilateral mastication

The dentition status was evaluated using the WHO criteria by trained and calibrated dentists. Third molars were regarded as nonfunctional teeth, and the final FTUs were defined as pairs of opposing functioning teeth (FST), which were composed of filled and sound teeth. The complete dentition comprised 12 FTUs, with two opposing premolars defined as 1 FTU and two opposing molars defined as 2 FTUs. In each subject, UM was calculated as the difference in the sums of the FTU scores on the two sides of the oral cavity: |sum of right-side FTUs − sum of left-side FTUs|. Based on the scores, the UM was classified into low (0), moderate (1 or 2), and high (more than 2).

### Assessment of confounding variables

Information on the study population including age, sex, household income, education level, BMI, waist circumference, noise exposure, smoking, and medical conditions was obtained from the KNHANES results. BMI was calculated by dividing the measured weight in kilograms by the square of the measured height in meters. Hypertension was defined as a systolic blood pressure of ≥140 mm Hg, a diastolic blood pressure of ≥90 mm Hg, or treatment with antihypertensive agents. Diabetes mellitus was defined as a fasting plasma glucose level of ≥126 mg/dL, treatment with oral hypoglycemic agents or insulin, or a diagnosis by a physician.

All of the participants were requested to fill in self-reported questionnaires to obtain information on certain items such as household income, education level, exposure to noise, smoking, and medical conditions, such as tinnitus. Smoking habits were categorized into current smokers and others. Education level was divided into up to high school or beyond high school. The lowest quartile of household income was considered a low income. Exposure to occupational, firearm, and recreational noise may be an important confounding factor in the association of masticatory function with hearing loss, and so the participants were divided into exposed or unexposed to such noise. Occupational noise exposure was defined as performing tasks for more than 3 months at noisy locations where people have to talk loudly to make themselves heard. Noise from a firearm was defined as exposure to a very loud sound, such as gunfire noise. Recreational noise exposure was defined as being more than 5 hours per week outside the workplace in locations where people need to speak loudly in order to talk with one another or the experience of using an earphone device in a noisy location. Tinnitus was defined when the subject answered ‘yes’ to each question of experiencing symptoms during the past year. Depression was defined when the subject answered ‘yes’ to a question of experiencing anxiety/depression in the EuroQoL questionnaires, which measures quality of life.

### Statistical analysis

The analyses were conducted using SPSS (version 23.0, SPSS, Chicago, IL, USA) with a significance cutoff of 0.05. All results were analyzed using a complex sampling plans and sampling weights of the KNHANES to provide nationally representative prevalence estimates.

Sociodemographic differences between participants with and those without hearing loss ascertained using the *t*-test for continuous variables and the chi-squared test for categorical variables. To identify the distribution of FST on each side of the oral cavity according to UM level, the number of FST was divided into total teeth, posterior teeth, and premolar and molar teeth on the right or left side. In addition, the average subjective masticatory ability was compared among different UM levels. Generalized linear models were used to compare mean values of FST and subjective masticatory ability among UM groups. Using multiple logistic regression analyses with adjustment for the covariates, the risk of hearing loss was estimated according to the UM level and was reported as odds ratios (ORs) and 95% confidence intervals (CI). The adjusted odds ratios (aORs) and their 95% CIs were calculated in multivariable logistic regression analyses. Sequential models were used to control for potential confounders. The primary model (model A) was adjusted for age, sex, household income, education level, a number of teeth, and unilateral mastication. Model B further adjusted for all variables in the primary model plus the health-related factors of diabetes mellitus, hypertension, current smoking, waist circumference, BMI, depression, and tinnitus. The final model (model C) further adjusted for all variables in model B plus the noise-exposure-related factors of occupational noise, firearm noise, and recreational noise.

## RESULTS

Overall, 678 of the included subjects (37.5%) had hearing loss (*P* < 0.001; Table 1), with the percentage of men (*n* = 379, 64.8%) being significantly higher than that of women (*n* = 299, 35.2%). The participants with hearing loss were older than the subjects with normal hearing (*P* < 0.001). The prevalence of hearing loss increased with age group. The subjects with hearing loss were more likely than were subjects with normal hearing to have a low income; low education level; obesity (BMI > 25); larger waist circumference (by about 3 cm); smoking behavior; exposure to occupational or firearm noise; chronic disease, such as diabetes or hypertension; and tinnitus. However, exposure to recreational noise and depression had no significant difference (*P* > 0.05). The rate of moderate and high UM was higher in subjects with hearing loss than in those with normal hearing.

**Table 1.** Sociodemographic, systemic health, noise exposure, and unilateral mastication (UM)^a^ data according to hearing loss (HL)^b^ status

Variables	All (*N*^c^ = 1,773, 100%)	HL (−) (*N*^c^ = 1,095, 62.5%)	HL (+) (*N*^c^ = 678, 37.5%)	*P*-value^d^
Age, years	50.41 (0.27)	47.42 (0.24)	53.39 (0.43)	<0.001
<45	514 (32.1)	419 (39.9)	95 (18.3)	
45–64	1079 (62.2)	648 (58.8)	431 (68.2)	
≥65	180 (5.7)	28 (1.2)	152 (13.6)	
Sex, male	733 (48.0)	354 (38.6)	379 (64.8)	<0.001
Low income, lowest quartile	174 (8.6)	49 (4.8)	125 (15.4)	<0.001
Education, <high school,	502 (26.1)	224 (19.9)	278 (37.3)	<0.001
Obesity, BMI >25	597 (35.5)	342 (34.0)	255 (38.3)	0.026
Waist circumference, cm	82.14 (0.28)	80.90 (0.36)	83.37 (0.40)	<0.001
Smoking, yes	283 (21.1)	158 (18.7)	125 (25.2)	0.026
Hypertension, yes	556 (28.8)	265 (23.9)	291 (37.7)	<0.001
Diabetes Mellitus, yes	154 (8.2)	65 (5.7)	89 (12.8)	<0.001
Tinnitus, yes	379 (20.5)	191 (17.1)	188 (26.7)	<0.001
Depression, yes	174 (9.0)	102 (8.6)	72 (9.7)	0.541
Noise exposure, yes				
Occupational noise exposure	236 (15.7)	113 (11.5)	123 (23.3)	<0.001
Firearm noise exposure	345 (23.7)	166 (19.7)	179 (30.9)	<0.001
Recreational noise exposure	117 (7.1)	32 (8.3)	32 (5.0)	0.075
Level of UM				<0.001
Low UM	1443 (81.8)	929 (85.2)	514 (75.8)	
Moderate UM	302 (16.6)	157 (13.9)	145 (21.6)	
High UM	28 (1.5)	9 (0.9)	19 (2.6)	

Data are expressed as numbers (weighted percentages) for categorical variables and mean (standard error) for continuous variables.

^a^UM, unilateral mastication was categorized into low, moderate and high according to side based FTU scores.

^b^HL, hearing loss defined as pure tone average >25 dB of thresholds assessed at 0.5, 1, 2, and 4 kHz.

^c^Unweighted number of participants.

^d^*P*-values were tested by the *t*-test for continuous variables and Pearson chi-square test for categorical variables.

Table 2 presents the distribution of the mean FST and subjective masticatory ability according to UM level. The number of total FST was highest in those with low UM (27.38), followed by moderate UM (26.15) and high UM (25.08). The same trend was shown in posterior teeth, with the number of FST lower in subjects with high UM than moderate UM (*P* < 0.05). Comparing the distribution of premolar and molar FST divided into right and left sides, subject with low UM had the highest number of premolar and molar FST on each side (*P* < 0.001). This pattern was consistent with the subjective masticatory ability using a 5-point Likert scale. Subject with low UM seemed to have significantly high ability in mastication (*P* < 0.001).

**Table 2.** Mean Functional Teeth (FST) and subjective masticatory ability according to unilateral mastication (UM) level

Variables	Low UM	Moderate UM	High UM
Number of total FST	27.38 (27.30–27.47)	26.15^a^ (25.92–26.37)	25.08^a^ (24.53–25.63)
Number of posterior FST	15.79 (15.74–15.84)	14.53^a^ (14.39–14.67)	13.50^a^ (13.18–13.82)
Number of right FST			
Premolar	3.98 (3.96–3.99)	3.91^a,b^ (3.87–3.96)	3.68^a,b^ (3.37–3.98)
Molar	3.92 (3.90–3.94)	3.33^a^ (3.23–3.42)	3.29^a,b^ (2.93–3.65)
Number of left FST			
Premolar	3.98 (3.97–3.99)	3.92^a^ (3.88–3.95)	3.80^a,b^ (3.65–3.95)
Molar	3.91 (3.88–3.94)	3.37^a^ (3.27–3.47)	2.72^a,b^ (2.25–3.20)
Subjective masticatory ability^c^	4.11 (4.04–4.18)	3.54^a^ (3.36–3.73)	3.57^a^ (3.04–4.10)

UM, unilateral mastication.

All variables were obtained from the complex samples general linear model and expressed as the mean (95% CI).

^a^Statistically significant difference compared with low UM, using Bonferroni tests, *P* < 0.001.

^b^Statistically significant difference compared with moderate UM, using Bonferroni tests, *P* < 0.05.

^c^Subjective questionnaire was formed using 5-point Likert scale: 1 = cannot chew at all; 2 = difficult to chew; 3 = cannot say either way; 4 = can chew some; 5 = can chew well.

**Figure 1.**
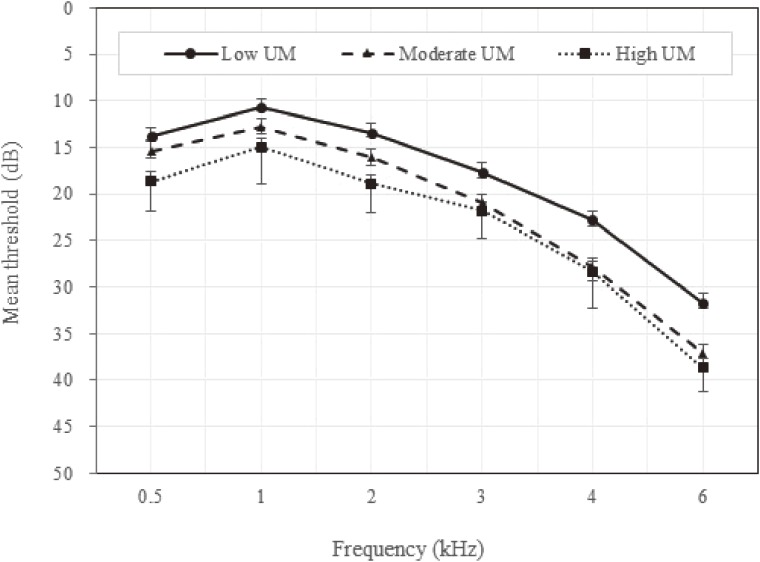
Mean pure-tone threshold values according to the unilateral mastication (UM) level

The mean pure-tone threshold gradually increased with frequency in all groups (Figure 1). The thresholds at all frequencies were higher in those with high UM than in those with moderate and low UM. Compared to low UM, there were significant mean differences in moderate UM and high UM, except at 0.5 kHz (*P* < 0.05, Bonferroni test, data not shown).

The results obtained for the covariate-adjusted logistic regression model for hearing loss are presented in Table 3 according to UM level. All models passed the Hosmer-Lemeshow goodness-of-fit test. When controlling for sociodemographic factors, the aOR for hearing loss was 3.12 (95% CI, 1.21–8.03) for high UM and 1.26 (95% CI, 0.87–1.83) for moderate UM relative to low UM. After adjusting for sociodemographic factors and health-related factors, high UM was associated with hearing loss (aOR 3.09; 95% CI, 1.13–8.42). This association remained in a fully adjusted model containing factors related to noise exposure (aOR 2.88; 95% CI, 1.12–7.46).

**Table 3.** Adjusted odd ratios (aORs) and 95% confidence intervals (CIs) for hearing loss according to unilateral mastication (UM) level

	Model A	Model B	Model C
Low UM	1	1	1
Moderate UM	1.26 (0.87–1.83)	1.27 (0.87–1.84)	1.29 (0.88–1.89)
High UM	3.12 (1.21–8.03)	3.09 (1.13–8.42)	2.88 (1.12–7.46)

UM, unilateral mastication.

Model A is adjusted for age, sex, house income level, education level, a number of teeth, and unilateral mastication. Model B is adjusted for all variables included in model A and further adjusted for diabetes mellitus, hypertension, current smoking, waist circumference, body mass index, depression, and tinnitus. Model C is adjusted for all variables in model B and further adjusted for noise at work, firearm, and recreational noise exposure.

Table 4 presents the crude and adjusted OR values of significant variables obtained for the final logistic regression model for evaluating hearing loss. The final model was selected based on regression model C, which included sociodemographic, health-related, and noise-exposure factors. The crude model revealed that all factors were significantly associated with an increased risk of hearing loss. However, the multivariable model showed that only sex, age, household income, education level, tinnitus, occupational noise, and UM were significantly associated with the risk of the hearing loss. The crude ORs for hearing loss in moderate and high UM were 1.75 (95% CI, 1.26–2.42) and 3.17 (95% CI, 1.22–8.21), respectively, compared with low UM. Meanwhile, when adjusting for all factors, only high UM was significantly associated with hearing loss (OR 2.88; *P* = 0.017).

**Table 4.** Final logistic regression model for evaluating the association with a hearing loss of >25 dB

Factors	Crude OR (95% CI)	*P*-value	Adjusted OR (95% CI)	*P*-value
Sex				
Female	1		1	
Male	2.93 (2.29–3.75)	<0.001	3.70 (2.53–5.40)	<0.001
Age group				
<44	1		1	
45–64	2.53 (1.87–3.43)	<0.001	2.23 (1.59–3.12)	<0.001
≥65	24.02 (14.03–41.13)	<0.001	15.66 (7.96–30.82)	<0.001
Household income (1,000 KRW)				
≥4,000	1		1	
3,000–3,990	1.31 (0.93–1.85)	0.117	1.28 (0.88–1.85)	0.094
2,000–2,990	1.60 (1.13–2.26)	0.008	1.40 (0.94–2.08)	0.007
<2,000	4.52 (2.91–7.03)	<0.001	3.13 (1.82–5.39)	0.003
Education level				
>high school	1		1	
≤high school	2.40 (1.84–3.15)	<0.001	1.67 (1.18–2.39)	0.003
Tinnitus				
No	1		1	
Yes	1.77 (1.33–2.35)	<0.001	1.60 (1.13–2.28)	0.003
Occupational noise				
No	1		1	
Yes	2.33 (1.61–3.37)	<0.001	1.92 (1.28–2.88)	0.004
Level of UM				
Low UM	1		1	
Moderate UM	1.75 (1.26–2.42)	0.018	1.29 (0.88–1.89)	0.662
High UM	3.17 (1.22–8.21)	0.001	2.88 (1.12–7.46)	0.017

CI, confidence interval; OR, odds ratio; UM, unilateral mastication.

Final logistic model is adjusted for age, sex, house income level, education level, a number of teeth, diabetes mellitus, hypertension, current smoking, waist circumference, body mass index, tinnitus, depression, noise at work, firearm, and recreational noise exposure, and unilateral mastication.

## DISCUSSION

To our knowledge, this is the first study to identify differences in hearing thresholds by UM level using large-scale epidemiologic data. In a representative sample of Korean adults aged 40 years and older who had taken part in KNHANES 2010–2012, the UM level was associated with reduced hearing loss, even after adjusting for confounding variables. Furthermore, we observed that the hearing threshold increased significantly with the UM level in this general population: the risk of hearing loss was about two-fold higher for high UM than for low UM.

Asymmetric FTUs can be used to classify the UM level and to estimate chewing ability. According to previous study, UM was associated with an imbalanced distribution of remaining teeth on the two sides of the mouth, and this can influence functional disturbances, such as an impaired chewing ability.^14^ Based on their findings, the UM level in the present study was estimated from asymmetric FTUs, which were found to be the single best predictor of masticatory performance.^15^^,^^22^^,^^23^ In particular, the loss of posterior teeth can result in shifting of the adjacent teeth that causes the potential collapse of bite support that in turn leads to a reduced masticatory force.^24^ This was supported by the present study finding that the high UM group—as defined using the FTUs for the posterior teeth—had the most impaired subjective masticatory ability and the smallest number of posterior FST among the three groups categorized according to UM levels (Table 2). It is, therefore, reasonable to evaluate the asymmetric level of FTUs when assessing unilateral mastication.

Some epidemiologic studies evaluated the associations between oral status and hearing loss.^9^^,^^25^ A previous study involving 1,156 United States veterans found a significant association between hearing decline and tooth loss when the latter was dichotomized into ≥17 and <17 teeth (OR 1.64; 95% CI, 1.24–2.17).^9^ In addition, it has been reported that the PTA is significantly higher in edentulous subjects.^10^^,^^25^ Consistent with these findings, the present study found that the risk of hearing loss was higher for high UM, for which the mean number of FST was lowest (25.08), compared to a mean number of FST of 27.38 for low UM (OR 2.88; 95% CI, 1.12–7.46). In another previous study, the mean difference in hearing loss between dentate and edentulous elderly was 5 dB (ranging from 4.9 to 8.6 dB), which was considered clinically significant.^25^ A direct comparison was not possible in the present study due to the absence of edentulous subjects, but the results of the present study were similar (Figure 1), with the 4-PTA increasing from 15.22 to 20.19 dB between low and high UM. It is meaningful that we attempted to extend the limited evidence available from male veterans^9^ and the elderly^25^ to the general population.

The results of the present study are also consistent with previous clinical studies finding that a unilateral chewing preference can affect the hearing ability and can be recovered by bilateral mastication treatment.^12^^,^^13^ These studies have suggested two possible mechanisms for UM—anatomical and neurological pathways. First, the mechanism may involve the anatomical pathway between the temporomandibular joint (TMJ) and middle ear. The abnormal tensile force of the discomalleolar ligament (DML), which arises from the malleus among auditory ossicles in the middle ear and runs to the medial retrodiscal tissue of the TMJ, can cause subjective hearing loss in patients with temporomandibular disorder.^26^^,^^27^ Excessive tensile force from UM may stretch the DML so as to adversely affect the efficiency of the auditory ossicles in conducting sound from the eardrum to the cochlea. Second, it can be explained via a neurological pathway for somatic stimulation. The trigeminal nerve responsible for sensation on the face converges on the dorsal cochlear nucleus (DCN) with cranial nerve 7, 9, and 10 and cervical nerve 2 and 3. Nonspecific stimuli in the DCN are, therefore, able to induce auditory problems. Somatosensory information resulting from muscle spasms is not produced by compression of nerves or blood vessels in the ear, but rather from the convergence in the DCN of sensory signals from muscle spindles in the head and neck with sound signals from the cochlea.^28^ Prolonged asymmetric exercise can induce muscle pain,^29^ and so facial pain from excessive UM can cause nonspecific stimulation and hearing problems. This supports the hypothesis that facial sensitivity problems associated with chewing can affect hearing ability.

This study postulated that the UM level, as measured by asymmetric FTUs, was associated with hearing loss. High UM clinically means that pairs of premolars and molars have been lost on one side of the mouth. It has been estimated that unilateral chewing occurs in 93% and 92% of cases of type-I and -II asymmetric shortened dental arches (SDAs), respectively.^30^ Asymmetric SDAs have a long side of the dental arches extending to the first molar, and so subjects with this feature compensate partly by chewing with the longest side. Therefore, our findings for high UM, which were based on asymmetry characterized by an FTUs score of more than 2, may reflect the effects on UM.

The important strengths of this study include the use of data from a large-scale population and consideration of well-known risk factors for hearing loss. This study found that age was a strong confounder of risk factors. A logistic regression analysis revealed a strong association between elderly group (≥65 years) and hearing loss. The hearing loss was also significantly associated with being male; having a low income, low education, and tinnitus; and being subjected to occupational noise in the final model (Table 4). These findings are consistent with those of previous studies.^6^^,^^21^ In addition to its broad generalizability, this is the first study to have considered the influence of unilateral mastication on hearing ability using the nationwide data set. A few previous studies have used FTUs,^16^^,^^17^ but no study has examined the influence of UM using a side-based asymmetry score of FTUs. Consequently, asymmetric FTUs can explain an association with hearing loss.

Some limitations of this cross-sectional study should be considered. First, our findings were based on cross-sectional data, so they cannot reveal cause and effect relationships. Second, this retrospective study was designed to analyze pre-existing data, and so we were unable to assess problems of the occlusal condition, subjective UM habits, and conductive hearing threshold. Moreover, we excluded the subject with untreated dental caries, artificial teeth (implants, bridges, and dentures), and CPI score 4 in order to estimate UM level. In case of CPI score of 4, it refers to advanced periodontal disease, which could cause impaired mastication due to biting pain, mobility of teeth, and decline in biting force during mastication. Although CPI index teeth do not include premolars, it can represent an individual’s periodontal status, even when compared to the entire recording.^31^ Nevertheless, further analyses that includes these factors will need to account for our results.

In conclusion, this study has shown that the UM, measured by asymmetric FTUs, is associated with hearing loss and confirmed that the individual FTUs can be used to assess the level of UM. These results indicate that adults with a higher probability of unilateral chewing experience greater impacts on impaired hearing ability than those without unilateral chewing based on assessing differences in FTUs.
